# Pseudohyperkalemia in chronic lymphocytic leukemia and diabetic ketoacidosis

**DOI:** 10.1002/ccr3.7821

**Published:** 2023-08-22

**Authors:** Mahfujul Z. Haque, Aishah Nasir, Ramzan Judge

**Affiliations:** ^1^ Michigan State University College of Human Medicine Grand Rapids Michigan USA; ^2^ Temple University Philadelphia Pennsylvania USA; ^3^ Shore Medical Center Somers Point New Jersey USA

**Keywords:** hematology, ICU, nursing, pseudohyperkalemia

## Abstract

Pseudohyperkalemia can lead to inaccurate hyperkalemia diagnosis, inappropriate initiation of potassium‐lowering therapies, and overall unnecessary treatment possibly inducing iatrogenic hypokalemia. Patients with leukocytosis and thrombocytosis should raise clinical suspicion that hyperkalemic laboratory results in the absence of other traditional signs of hyperkalemia may be indicative of pseudohyperkalemia. Here we present a case of severe leukocytosis with chronic lymphocytic leukemia (CLL) found to have critically elevated potassium levels on admission to the intensive care unit (ICU). The patient was also diagnosed with diabetic ketoacidosis (DKA) at admission, requiring an increased frequency of electrolyte monitoring. The events leading to the prompt recognition of pseudohyperkalemia in this patient will be delineated alongside our recommendations for revising the institutional protocol to avoid false hyperkalemia diagnoses in patients with CLL.

## Graphical abstract

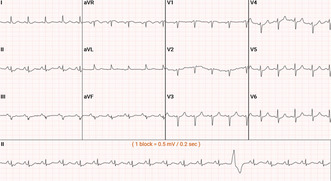

## 1 INTRODUCTION

Pseudohyperkalemia in the presence of chronic lymphocytic leukemia (CLL) and other forms of extreme leukocytosis (white blood cell [WBC] over 100,000 cells/μL) has been well documented. It is defined as a high serum potassium level unreflective of the true in vivo stores. It should be considered when there is a difference between the serum and plasma potassium of more than 0.4 mmol/L.^1^ In settings where the patient presents with leukocytosis, the electrocardiogram (EKG) remains normal and other pharmacological causes are ruled out, it is reasonable to suspect that pseudohyperkalemia.^2^ The pathophysiology resulting in this phenomenon is believed to be due to leukemic states causing fragility of WBCs, leading to increased cell lysis and resulting in intracellular release of potassium.^3^ Further contributing to the reporting of elevated potassium include both mechanical and chemical components of the original sample collection. Pneumatic tube transport of blood draws, prolonged incubation time of samples, excessive centrifugal source in specimen processing, and incomplete drying of ethanol containing antiseptics before venipuncture have all been noted to cause an increase in the frequency of hyperkalemic lab results.^4^

## 2 CLINICAL PRESENTATION

**FIGURE 1:**
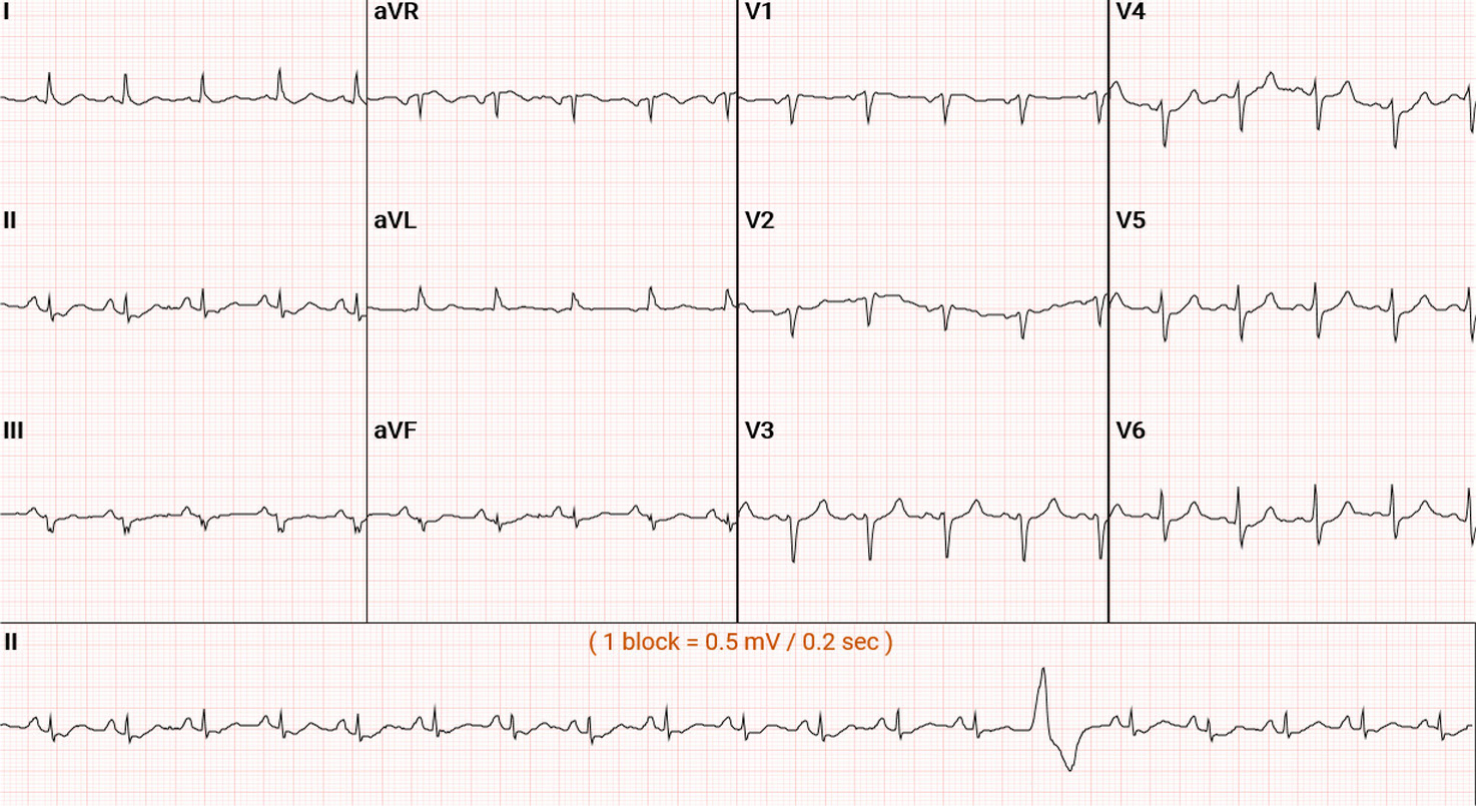
EKG on admission. EKG showed the patient to be in sinus tachycardia with ventricular premature complexes, biatrial enlargement, and an elevated QTc interval of 530.

A 57‐year‐old male presented to the emergency department for shortness of breath, having tested positive for coronavirus disease 2019 (COVID‐19) several days prior. He had a past medical history of hypertension, hyperlipidemia, diabetes mellitus, hypothyroidism, and chronic lymphocytic leukemia. After an initial blood draw in the emergency department, he was found to have diabetic ketoacidosis (DKA) and leukocytosis, with a WBC count of 311.87 k/μL. This initial blood draw, which was sent to the hospital laboratory via the pneumatic tube system and then centrifuged, resulted in a potassium level of >9.2 mmol/L (normal: 3.5–5 mmol/L). Although the patient had a normal EKG and lacked other signs or symptoms of hyperkalemia, he still received treatment for hyperkalemia, which included calcium gluconate, insulin, dextrose, and bicarbonate (see Figure 1). One hour after receiving hyperkalemia treatment, his potassium level was rechecked following the same procedure as previous draws and found to be 8.6 mmol/L. While still in the emergency department, two different whole blood samples were taken 1 h apart using the i‐Stat® Analyzer. This device is a portable analyzer for point‐of‐care blood analysis. These samples resulted in potassium levels of 5.5 mmol/L followed by 5.1 mmol/L. Once admitted to the ICU, the patient's subsequent potassium levels, which were all drawn and transported to the laboratory similar to that of the initial labs, were noted to be elevated at 6.1 mmol/L and 7.3 mmol/L. The hospital staff were instructed to walk the blood samples up to the laboratory rather than using the pneumatic tube system, and the potassium levels appeared to be normal by the second day of admission. Uric acid levels drawn from the same blood samples followed the same pattern as potassium, displaying an initial increase followed by normalization. Pertinent laboratory value trends can be seen in Table 1. Restricting the use of pneumatic tubes allowed for a more accurate reading of the patient potassium. Such a discrepancy raises the concern that the patient may not have true hyperkalemia. To consider all potential causes of this electrolyte elevation, the possibility of pseudohyperkalemia was explored.

**TABLE 1:** Trend of laboratory values.

Day of admission	Time of blood draw	Sample type	WBCs (4.5–11 k/μL)	Potassium (3.5–5 mmol/L)	Uric acid (3.5–7.2 mg/dL)
1	00:47	Routine blood draw^a^	311.87	>9.2	–
	02:51	Routine blood draw^a^	–	8.6	11.2
	03:11	Whole blood^c^	–	5.5	–
	04:32	Whole blood^c^	–	5.1	–
	09:29	Routine blood draw^a^	–	6.1	10.9
	12:15	Routine blood draw^a^	–	7.3	–
2	06:06	Routine blood draw^b^	–	4.5	–
	15:38	Routine blood draw^b^	316.69	–	–
3	05:50	Routine blood draw^b^	318.79	4.4	–
4	05:54	Routine blood draw^b^	361.92	5.2	5.9

^a^ Blood sample via pneumatic tube system.

^b^ Blood sample walked directly to the laboratory department.

^c^ Blood sample analyzed via i‐Stat® Analyzer.

## 3 DISCUSSION

Pseudohyperkalemia, or the elevation of serum potassium that does not represent the true in vivo stores, most commonly occurs in the hemolysis of the blood sample.^1^ Pseudohyperkalemia may occur in a series of pathologies including malignancies, hematological diseases, leukemias, leukocytosis, thrombocytosis, and as the result of pressurized centrifugal forces. Significant leukocytosis, which occurs in chronic lymphocytic leukemia, can lead to cellular weakening and, thus, a higher risk of cell lysis, as observed in this patient. An additional clinical phenomenon to consider when spurious hyperkalemia is expected is reverse pseudohyperkalemia. Reverse pseudohyperkalemia is present when the potassium concentration is elevated in plasma but normal in serum. The opposite is true in the aforementioned pseudohyperkalemia: potassium concentration is elevated in serum but normal in plasma (see Table 2).^5^, ^6^ At our institution, blood samples for patients who are considered in‐patient and staying in the hospital are collected via serum unless otherwise specified. Our patient's serum potassium level was elevated but normal in plasma. Both the American Family Physician guidelines for treating hyperkalemia and the American Heart Association advise to consider pseudohyperkalemia in the absence of other electrolyte abnormalities, normal whole blood potassium levels, and normal EKG. When suspected, it is recommended to repeat the sample collection as atraumatically as possible in tandem with obtaining both plasma and serum potassium values.^7^ In our patient, these factors along with the persistent elevation of potassium after treatment suggested pseudohyperkalemia as the cause. After identifying the possibility of pseudohyperkalemia, the patient blood samples were then walked up to the laboratory by hospital staff rather than using the pneumatic tube system. These resulted in normal potassium and uric acid levels.

**TABLE 2:** Differentiation of hyperkalemic conditions.

	Plasma	Serum
Hyperkalemia	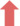 Elevated	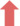 Elevated
Pseudohyperkalemia	Within limits	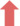 Elevated
Reverse pseudohyperkalemia	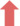 Elevated	Within limits

The pneumatic tube system is commonly used in hospitals to transport blood samples to the laboratory. It uses a pressure gradient to send tubes throughout the hospital and can reach speeds up to 25 feet per second or 18 miles per hour.^8^ The mechanical forces imposed on the blood sample from the tube system can cause cell lysis, especially in CLL patients.^9^ The tube system's high speed, rapid directional changes, and elevated pressure makes for a hazardous journey for the blood samples.^10^ A trial conducted by Streichert et al. focused on how blood samples transported to the laboratory either via pneumatic tube or hospital staff walk‐up could be affected by factors including temperature, humidity, pressure, and acceleration. No change was not noted between the two groups in any of these factors for 3‐axis acceleration. The study found significant changes in 3‐axis acceleration, which can be observed through differences in gravitational force. The pneumatic tubes were noted to have a maximum gravitational force of up to 15 g during transit and 9 g in those hand‐carried to the laboratory. It can be concluded that this force difference can significantly affect the blood samples and create higher rate of cell lysis. The authors also determined that lysis is more likely to occur as a result of changes in acceleration and the force on the cells rather than elevated acceleration alone.^11^ As the laboratory is on the sixth floor, the patient's samples had to travel at least four floors to arrive at its intended destination. This extensive travel time combined with the tube system's high speed and increased force could have further exacerbated the cell lysis in these blood samples.

These findings have been corroborated in case series literature concerning other CLL patients (see Table 3). Mansoor et al., highlighted a case of a 49‐year‐old male with CLL was reported to have a potassium level of 9.5 mmol/L on admission; however, when reverse pseudohyperkalemia was suspected considering a normal EKG in the face of extremely high potassium levels, a whole blood sample was collected which revealed a normal level of 3.7 mmol/L.^6^ Smalley et al., presented a case of a 79‐year‐old male with CLL was admitted to the ICU with potassium levels that continued trending higher (5.6 mmol/L at admission, 7.1 mmol/L the following morning)—yet his EKG remained without signs of hyperkalemia (i.e., peaked T waves or sine waves) and medications that could be causative were also ruled out. His serum potassium was obtained peripherally, tubed, and walked down to the lab, resulting in a reading of 3.2.^3^ While previous reports have discussed this phenomenon experienced by our patient, our patient also experienced concomitant DKA. This necessitated routine electrolyte analysis and for which the i‐Stat® was used. The utilization of whole blood samples via the i‐Stat® monitor can serve as a more efficient monitoring tool. When the reliability of the i‐Stat® is considered for use as a point‐of‐care device in evaluating plasma electrolyte levels, there exists a variety of literature informing our opinion about this function.^12^ In a study of critically ill pediatric patients, researchers concluded that the agreement between the i‐Stat® bedside and eventual laboratory values was satisfactory for potassium.^13^ Our hospital currently uses BD Vacutainer® PST™ gel and lithium heparin tubes, which exert additional pressure onto the blood sample prior to centrifugation. When the heparin in these tubes is centrifuged, it can cause cell destruction and potassium release as well.^14^ Whole blood can be analyzed on the floor using a device such as the i‐Stat® that provides immediate results. However, our hospital only allows whole blood testing in the emergency department. In the future, it would be beneficial to have a protocol in place to better detect potential pseudohyperkalemia. Such a protocol could include an alert for potassium levels in patients with CLL and elevated WBCs or the overall avoidance of using the pneumatic tube system for the blood samples of CLL patients. Lab abnormalities should be confirmed with a rapid whole blood sample in this patient population before initiating treatment.

**TABLE 3:** Summary of pseudohyperkalemia cases.

Case report	WBC (k/μL)	False potassium (mmol/L) level	True potassium (mmol/L) Level	Management
Semler et al. (2017)^13^	808	>14	4.7	Non‐heparinized tube, low RPM centrifugation
Rifkin (2011)^1^	273.9	6.8	2.7	Whole blood on arterial blood gas machine
Gujarathi et al. (2022)^14^	307.34	7.9	4.4	Whole blood with venous blood gas heparinized syringe
Alhaj et al. (2017)^15^	537	6.9	3.9	Whole blood with arterial blood gas heparinized syringe
Sindhu et al. (2011)^16^	276	7.5	3.5	Peripheral venous sample, Hand carried
Le et al. (2020)^17^	280	6.7	3.8	Point‐of‐care potassium
Kintzel et al. (2012)^18^	479	9.8	4.1	Heparinized tube and assayed immediately

## 4 CONCLUSION

Identifying incidences of pseudohyperkalemia and the pathological conditions it is associated with—namely thrombocytosis and leukocytosis, is imperative to avoid inappropriate treatment. According to our case study and associated research, CLL patients presenting with hyperkalemia should always be assessed via whole blood samples and institutional protocols should consider cautioning against the use of pneumatic tube systems in these patients. To avoid inaccurate hyperkalemia diagnosis and to prevent unnecessary treatment, we suggest a change in hospital protocol to reflect that serum potassium samples of individuals presenting with WBC in upward of 50–100 × 10^3^ k/μL, be obtained peripherally, tubed, walked to the lab, and directly communicated with the medical laboratory scientist team. Another appropriate alternative in ICU settings, especially when treating patients with CLL and DKA, requiring more frequent metabolic paneling, would be to increase the use of i‐Stat® for accurate bedside monitoring.

## AUTHOR CONTRIBUTIONS

**Mahfujul Z. Haque:** Conceptualization; data curation; formal analysis; funding acquisition; investigation; methodology; project administration; resources; software; supervision; validation; visualization; writing – original draft; writing – review and editing. **Aishah Nasir:** Conceptualization; data curation; formal analysis; funding acquisition; investigation; methodology; project administration; resources; software; supervision; validation; visualization; writing – original draft; writing – review and editing. **Ramzan Judge:** Conceptualization; data curation; formal analysis; funding acquisition; investigation; methodology; project administration; resources; software; supervision; validation; visualization; writing – original draft; writing – review and editing.

## FUNDING INFORMATION

The authors have not received any funding for this study.

## CONFLICT OF INTEREST STATEMENT

The authors declare that there is no conflict of interest.

## ETHICS STATEMENT

Our institution does not require ethical approval for reporting individual cases or case series.

## CONSENT

Written informed consent was obtained from the patient to publish this report in accordance with the journal's patient consent policy.

## Data Availability

The data that support the findings of this study are available from the corresponding author upon reasonable request.
